# Extranodal Extension as an Independent Prognostic factor in Laryngeal Squamous Cell Carcinoma Patients

**DOI:** 10.7150/jca.47700

**Published:** 2020-10-18

**Authors:** Zhihai Wang, Quan Zeng, Yanshi Li, Tao Lu, Chuan Liu, Guohua Hu

**Affiliations:** Department of Otorhinolaryngology, The First Affiliated Hospital of Chongqing Medical University, Chongqing 400016, China.

**Keywords:** laryngeal squamous cell carcinoma, head and neck squamous cell carcinoma, extranodal extension, prognosis

## Abstract

The presence of Lymph node metastasis with extranodal extension (ENE) is considered to be an important adverse prognostic factor for survival in patients with head and neck cancer. The aim of this study was to determine the prognostic significance of ENE in patients with laryngeal squamous cell carcinoma (LSCC). Three hundred and fifty-five patients with LSCC who underwent surgical resection and neck dissection were included. The status of cervical lymph node was classified into three groups: pathological negative nodal (pN-), pathological positive nodal without ENE (ENE-), and pathological positive nodal with ENE (ENE+). A total of 85 of 355 (23.9%) LSCC were pathological nodal positive, and ENE was detected in 22/355 (6.2%) patients. ENE was associated with drinking (*p*=0.005), T stage (*p*=0.000), tumor location (*p*=0.000), and differentiation degree (*p*=0.000). The number of lymph node metastasis in ENE+ group was associated with almost twice compared to ENE- group (*p*=0.005). The 5-year overall survival rates for patients in the pN-, ENE-, and ENE+ groups were 86.4±2.6%, 75.9±6.3%, and 53.7±12.7%, respectively (*p*=0.000). After adjusting for confounding variables, ENE+ was associated with more than five times the hazard of death than pN- cases (*p*=0.000), and more than twice the hazard of death than ENE- cases (*p*=0.036). Compared to N2-3/ENE- cases, N2-3/ENE+ cases had the poorest survival rate (*p*=0.013). ENE+ was associated with worse outcomes compared to pN - or ENE- status. ENE is an independent prognostic factor in LSCC, and could be an indicator of the need for adjuvant treatment.

## Introduction

Clinical tumor-node-metastasis (TNM) stage as the criterion has been more widely adopted in the therapeutic management of patients with malignant tumor. The lymph node staging system has been based on the number, size, and localization of metastatic positive nodes^1^-^3^. However, lymph node staging is sometimes not an adequate prognostic factor. A prognostic factor that can distinguish patients at high risk of death would be more helpful for adjuvant treatment strategy. Therefore, we need to consider the status of metastatic lymph node for adjuvant therapy following surgical treatment. The presence of ENE is considered to be an important adverse prognostic factor for patients with head and neck squamous cell carcinoma (HNSCC) who undergo primary surgery and has been demonstrated to be more associated with inferior locoregional recurrence and survival^4^-^7^. And ENE has been included in the category of metastatic lymph node in HNSCC^2^.

ENE of lymph node metastasis, which is defined as the expansion of tumor cells beyond the lymph node capsule into the perinodal adipose tissue, is considered to be one of the most important prognostic factors for survival in patients with several kinds of malignancies, including breast cancer, stomach, bladder, and HNSCC 7-10. Therefore, ENE is also involved in the American Joint Committee on Cancer (AJCC) eighth staging system for patients with vulvar cancer, penile cancer, and HNSCC^2^. However, there has been limited study of the prognostic role of ENE specifically in human LSCC. Recently, in the Sultan AbdÜlhamid Han Training and Research Hospital study of 81 patients with LSCC who underwent total laryngectomy and neck dissection, the rates of OS and two-year were respectively 69.2% and 46.2% in lymph node metastasis without ENE, and 61.5% and 38.5% in lymph node metastasis with ENE, and this modest difference was not statistically significant between the two groups for both OS and 2-year rates (p=0.440, p=0.341, respectively)^11^. The study concluded that ENE was not an independent prognostic factor in LSCC, likely related to a relative small number of patients and rather short-term following up.

Therefore, we analyzed retrospectively the outcomes of 355 LSCC patients who underwent surgical primary resection with neck dissection, which aimed to investigate the prognostic value of ENE in LSCC patients and to determine whether it can be a helpful guide for defining potential candidates for adjuvant treatment strategy.

## Materials and methods

### Patients

This study protocol was approved by the institutional review board of Institutional Animal Care Committee at Chongqing Medical University. As this study was a retrospective analysis of routine clinical data, participants' informed consent was waived by the institutional review board. The medical records of 355 patients with LSCC who underwent surgical resection and neck dissection at the First Affiliated Hospital of Chongqing Medical University (Chongqing, China) from March 2011 to December 2018 were retrieved from the center's database, and their clinical and histological characteristics were reviewed. None of these patients had ever received radiotherapy or chemotherapy. The eighth edition of AJCC TNM staging system was used for the staging of patients^2^. After the patients' initial evaluation, all important management decisions were made at a multidisciplinary team meeting including otorhinolaryngologist, radiologist and radiation oncologists. The therapeutic scheme was based on several factors, including TNM stage, patient preference, radiation-related morbidity, general performance status and so on. All patients in this study were underwent surgical resection of the primary tumor and neck dissection that was performed by the same multidisciplinary team. Each neck specimen was divided into levels by the surgeon and then sectioned in a routine manner and studied by the pathologist. The patients were divided into three groups by lymph node metastasis status: pathological nodal negative (pN-), pathological nodal positive without ENE (ENE-), and pathological nodal positive with ENE (ENE+). Indications for post-operative radiotherapy included advanced stage of the primary tumour, positive margin, pathological positive nodes and ENE. The average dose of irradiation was 56.8 gy (50-65 gy). In the case of positive margins and ENE, chemotherapy was added to post-operative radiation.

### Follow-up

Patients underwent a standardized postoperative follow-up schedule (including clinical examinations, electrolaryngendoscope, abdominal ultrasonography, neck and chest contrast-enhanced CT) every 3 months for the first postoperative year and every 6 months thereafter. If recurrence was suspected, patients underwent contrast-enhanced MRI, and/or PET-CT scan. The overall survival time was calculated from the day of operation until the time of death or final follow-up. The follow-up period ranged from 3 to 98 months (median, 47 months). The patients were followed up until death or the final follow-up date that was July 16, 2019.

### Statistical analysis

The data collection and statistical analysis were performed using SPSS version 21.0 software (SPSS Inc., Chicago, IL, USA). The χ^2^ test or Fisher's exact test was used to determine the incidence of metastasis and correlated factors. The overall survival rate was calculated by using the Kaplan-Meier method. Survival curves were compared between groups with the log-rank test. Postoperative follow-up data were available for all patients. The overall survival time was defined as the interval between the date of surgery and the date of the last consultation (censored) or date of death (event).

## Results

### Clinicopathologic features

From March 2011 to December 2018, the total of 355 patients who met the inclusion criteria for the present study consisted of 6 women (2.58%) and 349 men (97.42%), and the mean (SD) age of patients at the time of diagnosis was 60.15 ± 8.62 years (range 37-81 years). The follow-up period ranged from 3 to 98 months and the mean was 46.65 ± 23.08 months. Detailed clinical information was showed in Table 1. All 355 patients were classified according to the Eighth Edition AJCC^2^. In the univariate analysis, ENE was significantly associated with drinking, tumor location, T stage and differentiation degree, but no significant correlation was found in gender, age, and smoking. The rate of ENE increased obviously with the increase of T stage and decrease of differentiation degree, and there were also significantly difference in the rate of ENE between groups with glottis, supraglottic and subglottic carcinoma.

### Survival Analysis

**Figure 1:**
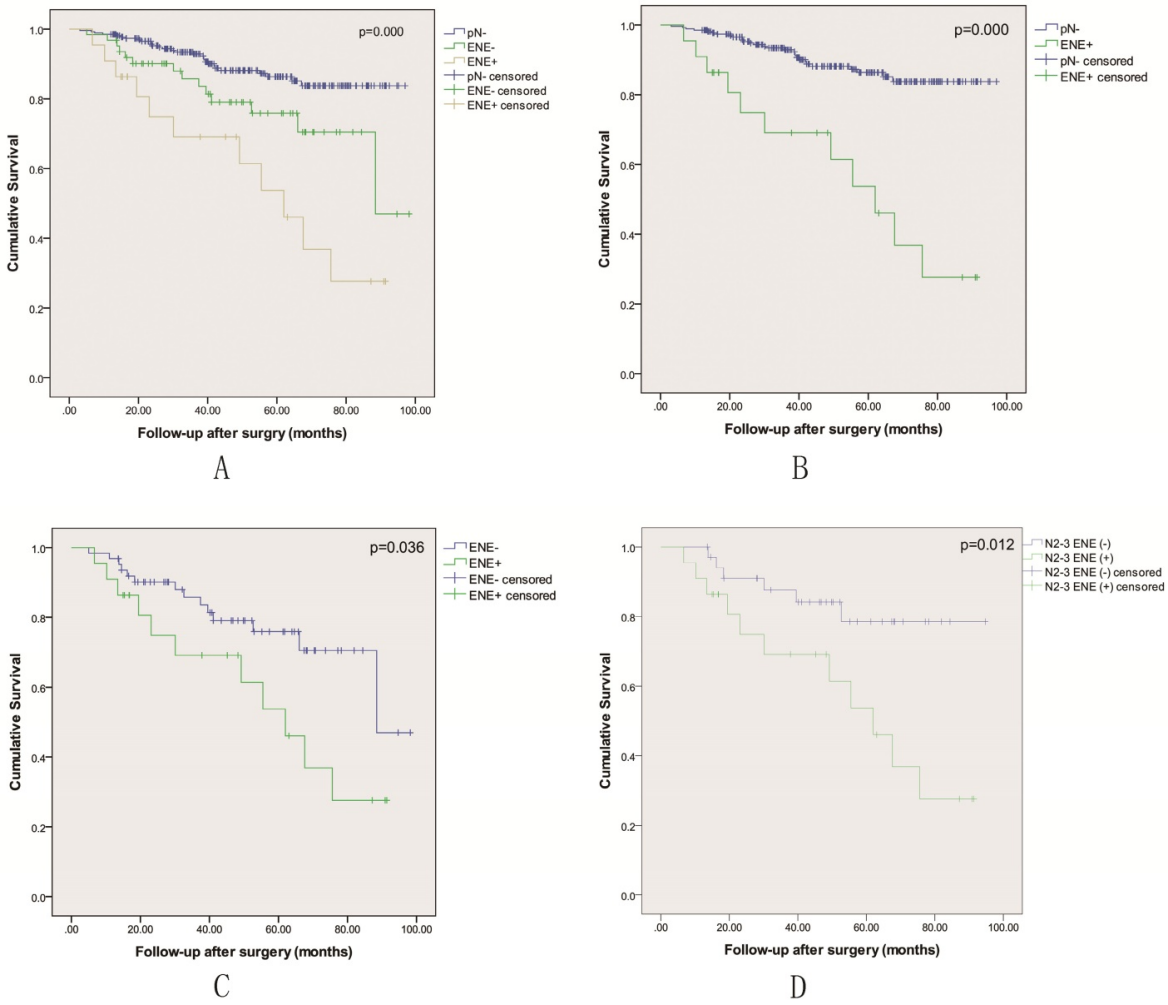
Kaplan-Meier overall survival curves based on the status of pathological nodal.

All 355 patients in the study were included in the survival analysis. There were 85 patients with pathological nodal positive, including 22 patients with lymph node metastasis with ENE. As shown in Table 2, the number of metastatic lymph node in ENE+ group was associated with almost twice compared to ENE- group (*p*=0.005). A multivariable analysis revealed that ENE was an independent prognostic factor for LSCC patients following surgical treatment (Table 3). Kaplan-Meier curves were used to evaluate the prognosis effect of LSCC patients. The 3-year and 5-year OS rates were 90.2± 1.7% and 82.3± 2.5%, respectively. When classified into three groups including pN-, ENE- and ENE+, the 5 year OS rate was respectively 86.4±2.6% vs. 75.9±6.3% vs. 53.7±12.7% (*p*=0.000), and the OS rate of the ENE+ group was the lowest (Figure 1A-C). The status of ENE further classified by AJCC 8th edition N stage was also significantly associated with survival (*p*=0.012), and N2-3 with ENE-positive disease had the lowest 5-year survival (Figure 1D).

### Sensitivity Analysis

Evaluation of survival according to lymph node metastasis status is shown in Table 3-5. In the multivariable analysis adjusted for confounding variables, ENE+ was associated with worse outcomes (HR = 5.290; 95% CI: 2.639-10.604) compared to pN- group (*p*=0.000) Table 3. Compared to ENE-, ENE+ was associated with more than twice the hazard of death (HR = 2.283; 95% CI: 1.032-5.047) (*p*=0.036) Table 4. Among cases that were N2-3, ENE-positive status was associated with more than three times risk of death (HR = 3.313; 95% CI: 1.222-8.982) compared with ENE-negative status Table 5 (*p*=0.013).

## Discussion

The prognosis of HNSCC following curative surgery is primarily based on primary site, resection margins, T stage, presence of metastatic lymph node, and disease stage. A potential prognostic factor which can identify a subgroup of HNSCC with poor prognosis that are most likely to helpful from concurrent adjuvant therapies, while minimizing the rate of death. Although the molecular biology of tumors has achieved great development in recent decades, the traditional clinical and pathological features of the tumor still play a vital role in biological behavior prognosis. Controlling the cervical lymph node metastasis is greatly important for determining the prognosis of HNSCC. Khoueir et al concluded that lymph node involvement affects the survival more than T stage in advanced LSCC^12^. If metastatic lymph node is ignored, the risk of regional recurrence or distant metastasis may increase greatly after surgery, which brings about the poor prognosis 13-15. In our study, when the patients were classified into three groups including pN-, ENE- and ENE+, the 5 year OS rate was respectively 86.4±2.6% vs. 75.9±6.3% vs. 53.7±12.7%, and the OS rate of the ENE+ group was the lowest. The result shows that lymph node status greatly affects the survival rate of LSCC patients following curative surgery.

The presence or absence of metastatic lymph node is considered to be one of the most vital prognostic factors for survival in HNSCC patients 16. Dünne et al. conducted a meta-analysis of 9 studies including 2573 patients with HNSCC to investigate the prognostic value of ENE, and concluded that the presence of ENE had a significant negative impact on survival (summarized odds ratio: 2.7, 95.0% confidence interval: 2.2-3.4) 17. Therefore, ENE is widely used as a marker predicting poor prognosis in patients with HNSCC 18-22. In the current study, 355 patients with LSCC who had negative margins after primary surgery were analyzed to determine the effect of ENE positivity on OS. We observed a significant association between ENE positivity and inferior OS, patients who had pathological positive ENE had a significantly poorer OS than ENE negative patients with lymph node metastasis, although the ENE+ group was treated with post-operative chemoradiotherapy, and the ENE is an independent prognostic factor on multivariable analysis. This is an important finding, because smaller single institution studies have failed to detect a significant association between ENE and prognosis in LSCC patients. In the Sultan AbdÜlhamid Han Training and Research Hospital study of 81 patients with LSCC who underwent total laryngectomy and neck dissection, The OS and two-year rate of 69.2% and 46.2% was observed in ENE positive patients versus 61.5% and 38.5% in ENE-negative patients, this modest difference was not statistically significant in this smaller cohort (p=0.440, p=0.341, respectively)^11^.

Since ENE was firstly defined by Bennett et al 23, a number of studies have reported that ENE is correlated to poor prognosis in patients with HNSCC. Moreover, ENE has been incorporated in the N stage in the AJCC 8^th^ edition. In our study, when stratifying all patients with N2-3 by ENE- and ENE+, cases that were ENE+ had the lowest 5-year survival, and ENE-positive status was associated with more than three times risk of death (HR = 3.313; 95% CI: 1.222-8.982) compared with ENE-negative status.

In conclusion, although our findings were influenced by the retrospective design of the study, including the lack of a randomized patient population and data from a single institution, it still demonstrates that ENE is an independent prognostic factor for LSCC patients following surgical treatment. Therefore, detailed pathological examinations are needed to evaluate the presence of ENE. A randomized controlled multicenter trial will be required to overcome the short of case and to prove whether the patients with LSCC who have ENE can benefit from adjuvant chemoradiotherapy.

## Figures and Tables

**Table 1:** Descriptive Statistics of All Cases by Extranodal Extension.

Term	Overall n=355	Node (-) n=270	ENE (-) n=63	ENE (+) n=22	χ^2^	*P*
Age						
≥60	197	147	36	14	0.78	0.677
<60	158	123	27	8		
Sex						
Male	349	265	62	22	0.43	0.809
Female	6	5	1	0		
Smoking					1.82	0.402
Yes	333	256	57	20		
No	22	14	6	2		
Drinking						
Yes	201	164	24	13	10.72	0.005
No	154	106	39	9		
Tumor site						
Glottic	254	220	25	9	59.12	0.000
Supraglottic	92	46	33	13		
Subglottic	9	4	5	0		
Differentiation*						
Well	209	174	26	9	42.67	0.000
Moderately	93	73	16	4		
Poor	50	20	21	9		
T stage						
T1	20	19	1	0	30.93	0.000
T2	145	125	18	2		
T3	141	98	30	13		
T4	49	28	14	7		

**Table 2:** The number of pathological positive nodal between ENE- and ENE+.

	n (Mean±SD)		*P* value
ENE-	2.29±1.42		
ENE+	4.27±1.35		0.005

**Table 3:** The multivariate survival analyses of all patients with LSCC.

	n (%)	HR (95% CI)	*P* value
Lymph node status			
pN-	270 (76.1)	1.00	
ENE-	63 (17.7)	2.134 (1.127,4.041)	0.017
ENE+	22 (6.2)	5.290 (2.639,10.604)	0.000
Tumor site			
Supraglottic	92 (25.9)	1.00	
Glottic	254 (71.5)	1.416 (0.732, 2.741)	0.301
Subglottic	9 (2.5)	0.752 (0.096, 5.876	0.786
Differentiation^*^			
Poor	50 (14.2)	1.00	
Moderately	93 (26.4)	0.709 (0.324, 1.550)	0.389
Well	209 (59.4)	0.597 (0.293, 1.215)	0.155
T stage			
T1	20 (5.6)	1.00	
T2	145 (40.8)	1.030 (0.237, 4.479)	0.968
T3	141 (39.7)	1.024 (0.231, 4.552)	0.975
T4	49 (13.8)	1.032 (0.213, 5.007)	0.969

CI = confidence interval; HR = hazardratio. ^*^3 patients could not be told the differentiation degree.

**Table 4:** Mutually Adjusted Hazard Ratios for ENE Among All Node-Positive Cases.

	n (%)	HR (95% CI)	*P* value
ENE-	63 (74.1)	1.00	
ENE+	22 (25.9)	2.283 (1.032,5.047)	0.036

CI = confidence interval; HR = hazardratio.

**Table 5:** Adjusted Hazard Ratios for Combined ENE and Nodal Stage Among All N2-3 Cases.

	n (%)	HR (95% CI)	*P* value
N2-3 ENE-	33 (60.0)	1.00	
N2-3 ENE+	22 (40.0)	3.313 (1.222,8.982)	0.013

## Abbreviations

ENE: extranodal extension
LSCC: laryngeal squamous cell carcinoma
pN-: pathological negative nodal
TNM: Clinical tumor-node-metastasis
HNSCC: head and neck squamous cell carcinoma
OS: over survival
AJCC: American Joint Committee on Cancer
